# Effect of glycemic control on cognitive function in patients with type 1 diabetes mellitus: a systematic review and meta-analysis

**DOI:** 10.1186/s13643-023-02433-9

**Published:** 2024-01-02

**Authors:** Wenting Hua, Zouxi Du, Tingting Lu, Limin Tian

**Affiliations:** 1 The First School of Clinical Medicine, Lanzhou University, Lanzhou, 730000 China; 2 Clinical Research Center for Metabolic Diseases, Gansu Province, Lanzhou, 730000 China; 3 Institution of Clinical Research and Evidence-Based Medicine, Gansu Provincial Hospital, Lanzhou, 730000 China; 4 Department of Endocrinology, Gansu Provincial Hospital, Lanzhou, 730000 China

**Keywords:** Type 1 diabetes mellitus, Cognitive function, Glycemic control, Systematic review, Meta-analysis

## Abstract

**Background:**

It is controversial whether the level of glycemic control in patients with type 1 diabetes mellitus (T1DM) correlates with reduced cognitive function. This study explored the influence of glycemic management quality on cognitive function in T1DM patients by examining the association between glycemic control level and impaired cognitive function.

**Methods:**

The electronic databases PubMed, Embase, Cochrane Library, China National Knowledge Infrastructure, China Science and Technology Journal database, Wanfang database, and China Biology Medicine disc database were systematically searched to identify eligible studies published before January 2023. Search, selection, and data extraction were performed by two independent reviewers. RevMan 5.4 software was used for meta-analysis, and standardized mean difference (SMD) between groups was calculated.

**Results:**

Six studies involving 351 patients with T1DM were included in this study. Compared with T1DM subjects with good glycemic control, those with poor glycemic control performed worse in full-scale intellectual quotient (*P *= 0.01, SMD = −0.79, 95%CI = −1.42 to −0.17), but no significant differences were observed in verbal intellectual quotient (*P *= 0.08, SMD = −1.03, 95%CI = −2.20 to 0.13), memory (*P *= 0.05, SMD = −0.41, 95%CI = −0.82 to 0.00), and attention (*P *= 0.23, SMD = −0.26, 95%CI = −0.69 to 0.16).

**Conclusions:**

T1DM patients with suboptimal glycemic control may have a worse cognitive function, mainly focusing on the full-scale intellectual quotient. The current study highlights the significance of maintaining satisfactory glycemic control in T1DM patients to improve their health status and quality of life. Standardized tests should be employed in clinical neuropsychological practice to provide early and complete cognitive assessment of individuals with poor glycemic control.

**Systematic review registration:**

The study protocol has been registered in the PROSPERO database (CRD42023390456).

**Supplementary Information:**

The online version contains supplementary material available at 10.1186/s13643-023-02433-9.

## Introduction

Diabetes mellitus (DM) is a chronic metabolic disease caused by insufficient insulin secretion or deficiencies in insulin action or both, mainly characterized by hyperglycemia [1]. Based on the latest survey by the International Diabetes Federation (IDF), 537 million adults suffer from DM, which is predicted to rise to 700 million in 2045 [2]. DM is classified as type 1 diabetes mellitus (T1DM), type 2 diabetes mellitus (T2DM) depending on various pathogenesis, and gestational diabetes mellitus, etc. [3]. T1DM is an autoimmune disease leading to the destruction of β cells, absolute insulin deficiency and hyperglycemia, with approximately 5–10% of all diabetes cases [3]. However, T1DM can occur at any age, mainly involving children and adolescents, of which more than 1.2 million are living with T1DM [2]. Currently, insulin replacement is still considered as the best therapy for patients controlling blood glucose in T1DM [4].

Cognition function is an advanced neurological function and an important ability in the brain, which acquiring knowledge and understanding through thought, experience, and senses [5]. With the improvement in medicine, a growing body of evidence suggests that various pathologies or diseases can impair cognitive function [6, 7]. One of the most common diseases in the field of endocrine and metabolic diseases, DM has been shown to cause cognitive impairment in the brain. A longitudinal cohort study found that T2DM patients had cognitive decline in executive function, concentration, and attention [7]. It is suggested by some clinical studies hyperglycemia is a potential risk factor for mild cognitive impairment (MCI) or Alzheimer’s disease, and MCI patients are more likely to develop dementia than the general population [8]. Moreover, reports have confirmed that people with diabetes have twice the risk of developing dementia as people without diabetes [9]. This may be related to the fact that the hyperglycemia can increase the accumulation of β-amyloid in brain lesions, aggravating oxidative stress, neuroinflammation and mitochondrial dysfunction, and damage neuronal integrity [10]. With significant improvements in survival for patients with T1DM [11], the problem of dementia associated with T1DM has attracted attention. Patients with T1DM perform worse than normal on neuropsychological tests in the areas of memory, learning, and executive function [12]. The results of one study showed that nearly half of patients with childhood or adult-onset T1DM had clinically significant cognitive impairment at an average age of 68 years [13]. Recent studies have suggested that T1DM patients with suboptimal glycemic control perform worse in terms of psychomotor speed, language, and overall cognitive performance [14]. Interestingly, Ohmann et al. proposed that there was no significant association between the level of glycemic control and brain cognitive function [15].

Therefore, this study conducted a systematic review and meta-analysis of the currently available research evidence to investigate the potential correlation between cognitive impairment and the quality of diabetes management in patients with T1DM by analyzing the association between glycemic control level and impaired cognitive function.

## Methods

The present review was conducted according to the Joanna Briggs Institute (JBI) methodology for systematic reviews of etiology and risk [16] and is reported according to the Preferred Reporting Items for Systematic Reviews and Meta-Analyses [17], and the checklist is shown in Additional file 1. The study protocol has been registered in the PROSPERO database (CRD42023390456).

### Eligibility and exclusion criteria

#### Eligibility criteria

Observational studies that met the following "PEO" structure were included.

 Participants (P): Patients with T1DM.

 Exposure of interest (E): The exposure of interest was the level of glycemic control. Patients were divided into good controlled and poorly controlled groups according to glycated hemoglobin (HbA1c) levels.

 Outcomes (O): Cognitive function. The study protocol included at least one measure of cognitive function, such as intelligence, memory, attention, psychomotor speed and so on.

#### Exclusion criteria

Duplicate literatures, reviews, animal studies, reviews, conference abstracts, academic articles, and non-Chinese or English publications were excluded.

### Search

The electronic databases PubMed, Embase, Cochrane Library, Web of Science, China National Knowledge Infrastructure (CNKI), China Science and Technology Journal database, Wanfang database, and China Biology Medicine disc database were systematically searched to identify eligible studies published before January, 2023. Synonyms of “type 1 diabetes,” “cognition,” and “glycemic control” were searched by combining subject headings (i.e., MeSH) and free text words, the detailed search strategy was described in Additional file 2.

### Study selection

After removing duplicate search results using EndNote X9 (Thomson Corporation, USA), the remaining articles were screened by two reviewers based on titles and abstracts. Then, the initially included articles were screened on the basis of full text to assess whether they met the inclusion criteria. The reference lists of the included articles were examined to identify any additional relevant literature. A third reviewer was consulted when two reviewers disagreed. Reasons for exclusion were recorded for all excluded literature.

### Data extraction

Two reviewers independently extracted the relevant data. Any disagreements were arbitrated by a third reviewer. For each included study, the following data were extracted: study characteristics (publication year, name of author, country), study design, sample characteristics (sample size, age, country, duration of diabetes), and raw scores of cognitive function tests, including means and standard deviations of the good and poor glycemic control samples.

This review classified cognitive function into more specific cognitive domains of intelligence, memory, and attention. In addition, some studies included cognitive tests that did not fit into any of these domains; these were classified into categories of other cognitive functions. Among the included literatures, one study [18] was divided into three groups according to HbA1c, and the standard formula see formula (1)–(3) [19] was used to combine the relevant research indicators $\overline{x}\pm$s in two groups with 7.5% as the boundary.

1 $$N=N1+{N}_2$$

2 $$M=\left(N1M1+N2M2\right)/\left(N1+N2\right)$$

3 $$SD=\sqrt{\frac{\left({N}_1-1\right){SD}_1^2+\left({N}_2-1\right){SD}_2^2+\frac{N_1{N}_2}{N_1+{N}_2}\left({M}_1^2+{M}_2^2-2{M}_1{M}_2\right)}{N_1+{N}_2-1}}$$

Equations (1)-(3), *N* is the combined sample size, and N_1_ and N_2_ are the sample size of the two groups; *M* is the combined mean, and M_1_ and M_2_ are the mean of the two groups; SD is the combined standard deviation, and SD_1_ and SD_2_ are the standard deviation of the two groups.

### Assessment of risk of bias

Risk of bias for the included studies was assessed by two reviewers using the critical appraisal tool provided by the JBI. The tool included a total of eight items, and each item was rated as “low risk,” “high risk,” or “unclear” [16].

### Synthesis of evidence

Meta-analysis was performed when two or more studies with similar study designs, and outcome measures could be combined. The meta-analysis was conducted using RevMan 5.4 [20]. Regarding cognition, studies were grouped by cognitive domains and standardized mean difference (SMD) were calculated. All measures were reported with the 95% confidence interval (CI). The heterogeneity between studies was assessed using the *I*^2^ statistic, and an *I*^2^ value > 50% was considered as highly heterogeneous and a random effects model would be used. In the case of high heterogeneity, sensitivity analyses were performed by excluding one study at a time to explore whether individual studies accounted for heterogeneity. *P *< 0.05 was considered statistically significant.

For *I*^2 ^> 30% and more than 5 studies included, the prediction interval (PI) from the random-effects meta-analyses is used. It predicts the potential underlying effect in a new study that is different from the average effect from the meta-analyses [21].

### Quality of the evidence

The Grading of Recommendations Assessment, Development and Evaluation (GRADE) criteria were used to assess the quality of the evidence for each outcome [22].

## Result

### Study selection

**Fig. 1:**
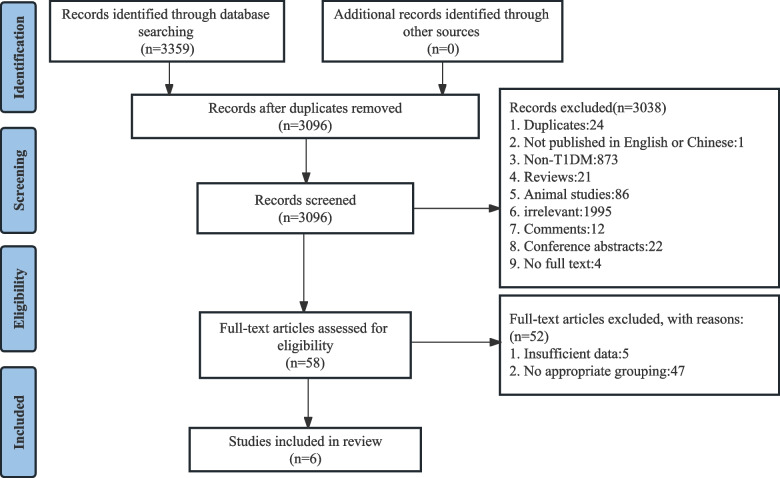
Flow diagram of the study selection

A total of 3359 records were retrieved. After eliminating duplicates, a screening of the remaining 3096 studies was performed based on titles and abstracts, of which 3038 were excluded. The remaining 58 articles were subsequently read in full text. Ultimately, six studies were eligible for the meta-analyses. The PRISMA flow chart of study selection is shown in Fig. 1.

### Study characteristics

A total of six studies [15, 18, 23–26] were included, all of which were cross-sectional. They were published from 2009 to 2018, and the study areas were distributed in Austria [15, 26], China [25], Poland [18], Egypt [24], and Germany [23]. Studies provided data on the full-scale intellectual quotient (FSIQ) [18, 24–26], verbal intellectual quotient (VIQ) [18, 24], memory [15, 18, 23–26], and attention [18, 23]. According to HbA1c grouping, 8.0% was used as the cutoff in two studies [15, 26], and 7.5% was used in the rest [18, 23–25]. Five studies included adolescent patients [15, 18, 24–26], and one study included adult patients [23]. A total of 351 patients with T1DM was involved, and two of the studies had small sample sizes [23, 26]. The basic characteristics of the included studies were shown in Table 1.

**Table 1:** Characteristics of the included studies

Study	Country	Sample size Group 1/Group 2	Age (years) Group 1/Group 2	HbA1c (%) Group 1/Group 2	Duration of diabetes Group 1/Group 2	Assessment of cognition	Study type
Ohmann 2009 [15]	Austria	36/34	14.03±2.55/ 15.56±1.93	6.91±0.54/ 9.29±0.40	4.39±3.10/ 7.49±3.16 years	WISC-III, WAIS-R	cross-sectional
Zihl 2010 [23]	Germany	16/12	34.20±10.30/ 30.50±12.80	6.80±0.50/ 9.40±1.90	164.20±101.40/ 197.80±146.80 months	DST, WMS-R	cross-sectional
Kaufmann 2012 [26]	Austria	15/15	14.70±4.10/ 13.90±3.90	7.40±0.50/ 9.20±1.20	6.80±4.50/ 4.40±3.10 years	WISC-III, WAIS-R, SWM	cross-sectional
Abo-el-Asrar 2016 [24]	Egypt	17/33	11.18±1.85/ 12.06±1.97	6.63±0.21/ 9.35±1.69	5.47±1.55/ 6.91±2.11 years	BVRT, WISC	cross-sectional
HE 2018 [25]	China	32/73	13.20±3.35/ 11.85±3.37	6.60±0.69/ 9.70±1.93	3.11±2.96/ 2.30±2.78 years	WISC-RC, WAIS-RC	cross-sectional
STANISŁAWSKA-KUBIAK 2018 [18]	Poland	21/47	11.39±2.66/ 13.35±2.31	-	4.50±2.99/ 5.80±2.96 years	WISC-RC, Brickenkamp’s and Zillmer’s d2 test, The trial of 10 words	cross-sectional

*WISC-III* Wechsler Intelligence Scale for Children-III, *WAIS-R* Wechsler Adult Intelligence Scale-Revised, *WISC-RC* Wechsler Intelligence Scale for Children-Chinese Revision, *WAIS-RC* Wechsler Adult Intelligence Scale-Chinese Revision, *BVRT* Benton Visual Retention Test, *DST* Digit Symbol Test, *WMS-R* Revised German version of the Wechsler Memory Scale, *SWM* Spatial Working Memory, Group 1 Good-controlled group, Group 2 Poor-controlled group

### Risk of bias

**Fig. 2:**
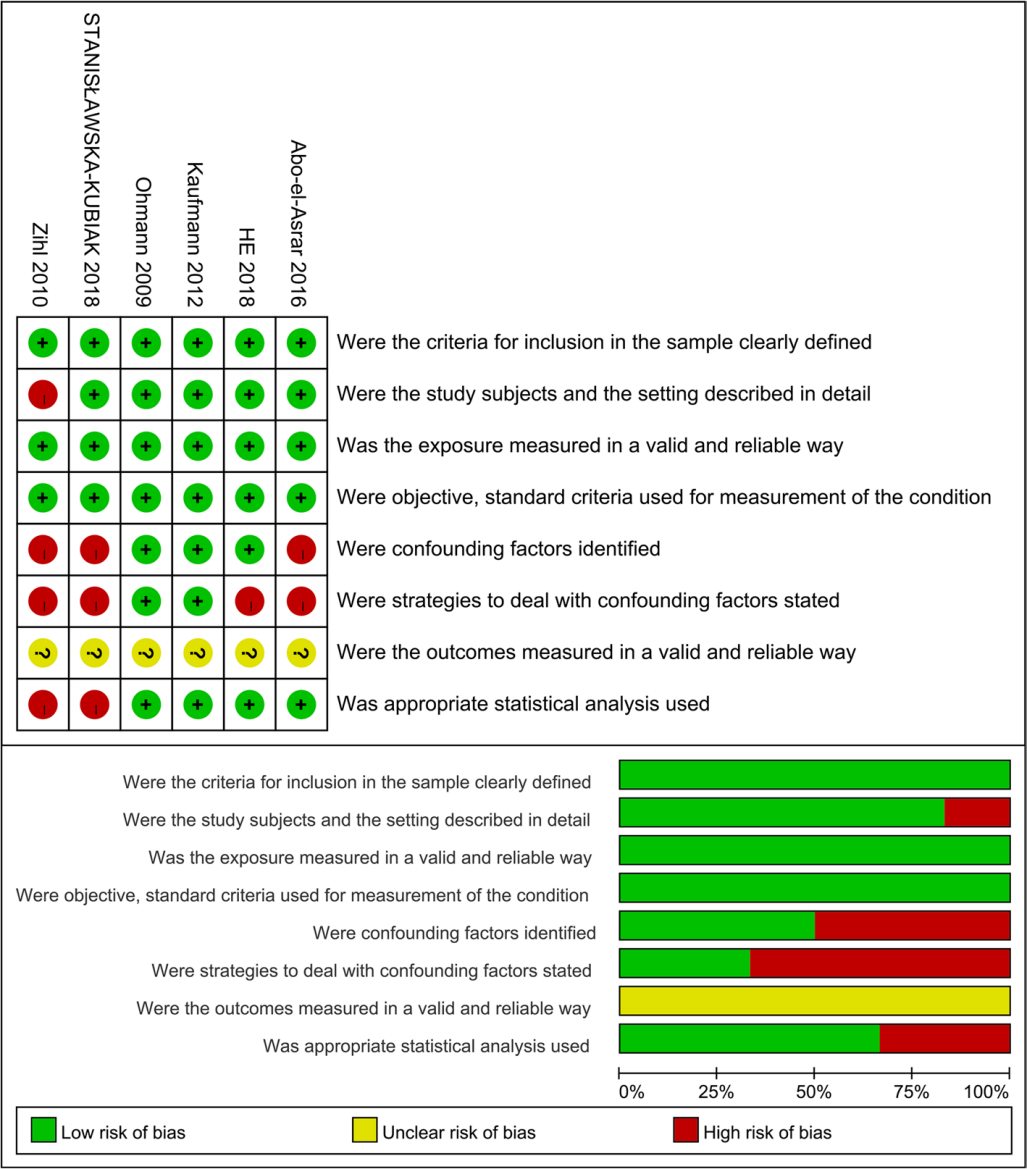
Risk of bias of included studies

Almost all studies had clear inclusion and exclusion criteria for participants. However, there is an unclear risk of bias in the measurement of outcomes due to some subjectivity in using scales to assess cognitive function. The risk of bias in the included studies was depicted in Table 1 (Additional file 3) and Fig. 2.

### Association between glycemic control and cognition in patients with T1DM

#### Full-scale intellectual quotient

**Fig. 3:**
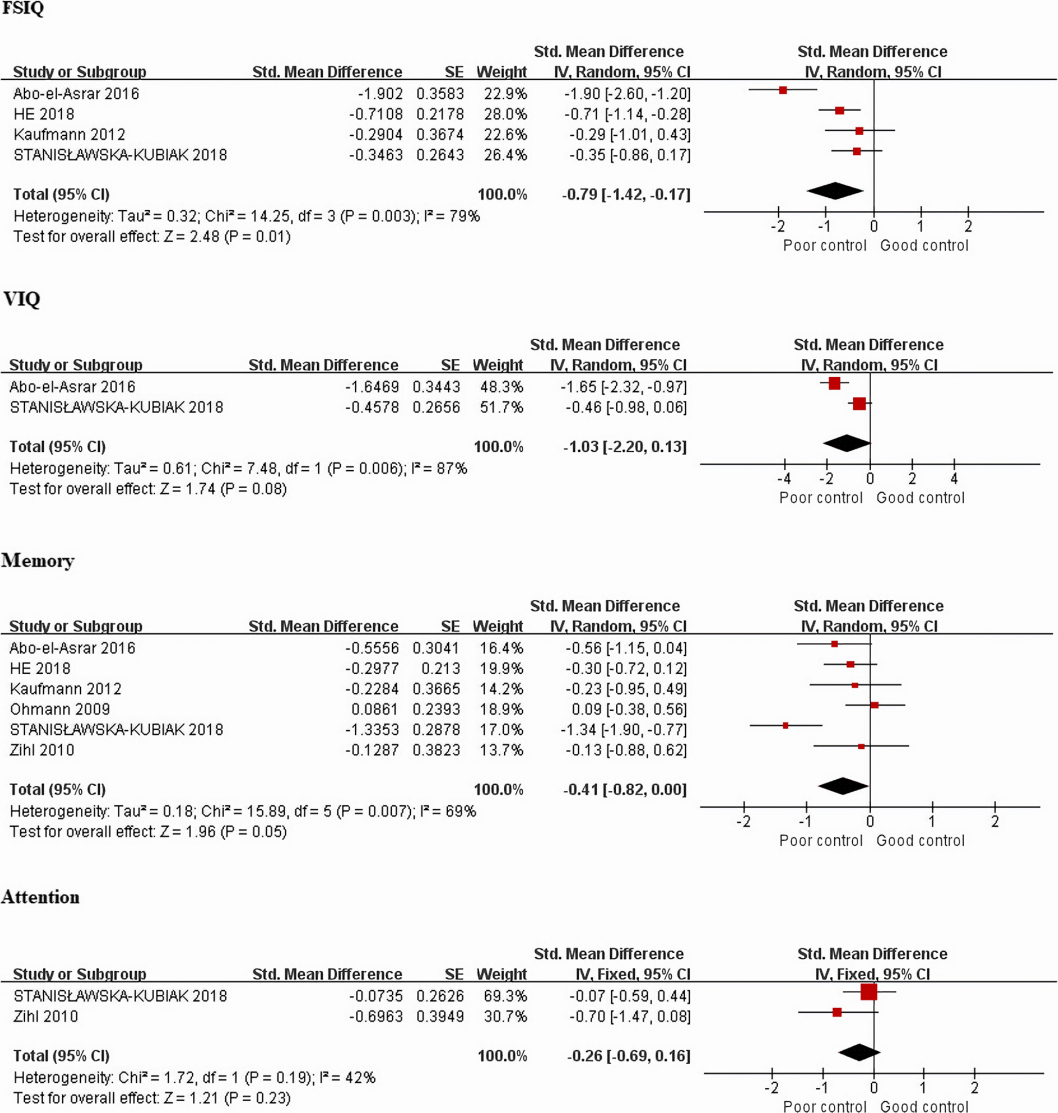
Forest plots of the relationship between glycemic control and cognitive outcomes

Four studies [18, 24–26] examined the FSIQ. Meta-analysis showed that patients with poor glycemic control scored lower on the FSIQ compared to those with good glycemic control (*P *= 0.01, SMD = −0.79, 95%CI = −1.42 to −0.17, *I*^2 ^= 79%) (Fig. 3).

#### Verbal intellectual quotient

Two studies [18, 24] examined the VIQ. The results of meta-analysis showed that there was no significant difference between the two groups (*P *= 0.08, SMD = −1.03, 95%CI = −2.20 to 0.13, *I*^2 ^= 87%) (Fig. 3).

#### Memory

Six studies [15, 18, 23–26] examined memory. Meta-analysis found that patients with poor glycemic control scored similarly on memory compared to those with good glycemic control (*P *= 0.05, SMD = −0.41, 95%CI = −0.82 to 0.00, *I*^2 ^= 69%, PI = −1.76 to 0.94) (Fig.1 (Additional file 3) and Fig. 3 ).

#### Attention

Two studies [18, 23] examined attention. Meta-analysis showed that patients with poor glycemic control scored similarly on attention compared to those with good glycemic control (*P *= 0.23, SMD = −0.26, 95%CI = −0.69 to 0.16, *I*^2 ^= 42%) (Fig. 3).

### Sensitivity analyses

For the FSIQ, the heterogeneity was 0% after removing Abo-el-Asrar’ study [24] (*P *< 0.001, SMD = −0.52, 95%CI = −0.82 to −0.22), and for the memory, heterogeneity was fully explained by STANISŁAWSKA-KUBIAK’ study [18] (*P *= 0.09, SMD = −0.21, 95%CI = −0.46 to 0.03, *I*^2 ^= 0%). After exclusion, a meta-analysis of the remaining studies showed that the results changed in the same direction as before exclusion.

### Grading of the evidence

The summary of the GRADE assessment for each outcome was shown in Table 2. The evidence certainty was very low for all outcomes assessed in this systematic review, starting with a low rating because the data were from observational studies, and the certainty of the evidence was further downgraded for inconsistency or imprecision.

**Table 2:** Quality of evidence according to GRADE approach

Outcome indicator	No. studies (participants)	SMD (95%CI)	Bia of risk	Inconsistency	Indirectness	Imprecision	Publication bias	Evidence quality
FSIQ	4 (253)	−0.79[−1.42, −0.17]	Not serious	Serious ^a^	Not serious	Not serious	None ^c^	Very low
VIQ	2 (118)	−1.03[−2.20, 0.13]	Not serious	Serious ^a^	Not serious	Serious ^b^	None ^c^	Very low
Memory	6 (351)	−0.41[−0.82, 0.00]	Not serious	Serious ^a^	Not serious	Not serious	None ^c^	Very low
Attention	2 (96)	−0.26[−0.69, 0.16]	Not serious	Not serious	Not serious	Serious ^b^	None ^c^	Very low

*GRADE* Grading of Recommendations Assessment, Development, and Evaluation, *SMD *standardized mean difference, *CI *confidence interval; Explanations: a, greater heterogeneity in combined results, I^2^ > 50%; b, the optimal information size was not achieved in the meta-analysis; c, publication bias could not be investigated due to the small number of included studies (<10)

## Discussion

In recent years, the impact of T1DM on the brain cognitive function has received extensive attention. However, the relationship between glycemic control level and cognitive function in T1DM patients is still unclear according to current research. In this study, a meta-analysis was conducted to compare the cognitive function of T1DM patients with satisfactory and suboptimal glycemic control. The neuropsychological results showed that poor glycemic control had a significant adverse effect on FSIQ. Although no statistically significant correlation was found with VIQ, memory, and attention, the combined estimates pointed to a negative association. These findings are consistent with previous studies showing that suboptimal glycemic control has a negative impact on cognitive function in patients with T1DM [27–29].

Patients with T1DM have large fluctuations in blood glucose levels due to absolute insulin deficiency and lifelong dependence on insulin therapy [30]. Therefore, satisfactory or stable glycemic control is particularly important. In a large cohort study, patients with T1DM who had a mean HbA1c concentration of less than 7.4% performed significantly better on tests of the speed of thought and visual-motor integration than those with mean HbA1c concentration of more than 8.8% [28]. As the data were not available, we were unable to merge to obtain consistent results. It has been reported that suboptimal glycemic control can negatively affect cognitive function in young patients with T1DM [27]. In a prospective longitudinal study, cognitive decline in patients with early diagnosed T1DM was associated with chronically high HbA1c levels [31]. Mauras and colleagues found that brain volume and cognitive scores were inversely associated with HbA1c levels in children with T1DM [32]. At the same time, previous research has shown that patients with well controlled diabetes did not have a significantly increased risk of cognitive impairment compared with healthy individuals without diabetes. Diabetes and glycemic control were strongly associated with incident MCI in people with normal cognition at baseline [8]. However, Ohmann et al. found that cognitive function was significantly impaired in children and adolescents with T1DM, and it was not associated with the quality of glycemic control [15]. Preclinical animal models of T1DM have also highlighted that hyperglycemia increases the risk of cognitive impairment. In the streptozotocin (STZ) model, chronic hyperglycemia tends to result in decreased performance on behavioral tests of spatial learning and memory (e.g., Morris water maze, Y-maze, and conditional active avoidance) and visuospatial object recognition memory [33, 34]. Biessels et al. demonstrated that the administration of insulin effectively mitigated spatial learning and synaptic plasticity impairments in STZ-induced diabetic rats, but only when insulin treatment was initiated immediately after the induction of hyperglycemia [35]. This also suggests the importance of early standardized treatment to reduce exposure to hyperglycemia.

The current research supports the concept that insufficient glycemic control increases vulnerability to cognitive decline, and there are plausible mechanisms that can explain the influence of suboptimal glycemic regulation on cognitive function. First, poor glycemic control means that the body is in a state of hyperglycemia for a long time, and in this state, the cells of the body are susceptible to stress (including oxidative stress and endoplasmic reticulum stress) and inflammatory response, which will lead to cell dysfunction, thereby impairing the normal function of brain cells. Second, an increase in glucose levels can damage the lining of blood vessels, these injuries may lead to cerebral vascular diseases, such as cerebral hemorrhage and cerebral infarction. Third, in a state of long-term hyperglycemia, brain neurons are vulnerable to damage and death [36–39]. These structural changes in the brain caused by cellular and vascular alterations resulting from chronic hyperglycemia may underlie the pathophysiology of cognitive impairment. In functional magnetic resonance imaging, higher HbA1c levels are associated with lower brain activation, This may reflect the upregulation of glucose transport in the brain [40]. Data from the current study suggest that controlling blood glucose within the near-normal range may reduce the risk of cognitive decline in patients with diabetes. When blood glucose control is suboptimal and the brain is in a hyperglycemic state for a long time, it can affect cognitive function. Cognitive function is particularly important for glycemic control in patients with T1DM, as poor cognitive function may affect patients’ understanding and implementation of self-management, as well as their adherence to medication use and diet control, leading to suboptimal glycemic control, which in turn affects cognitive function, forming a vicious circle.

The strength of this review is that it follows the best practice guidelines for systematic reviews and meta-analyses, combining the available research evidence and clarifying the conclusions. To our knowledge, few studies have reported the relationship between glycemic control level and cognitive function in T1DM patients. There are several limitations that should be acknowledged. First, in present study, the included studies were cross-sectional and lacked high-quality longitudinal studies. Second, HbA1c is the best response to glycemic control level, but does not reflect blood glucose fluctuations and the risks associated with extreme hypoglycemia and hyperglycemia. Third, due to the limitations on the number of studies included, we were unable to do subgroup analyses to assess the effect of confounding factors (e.g., age, disease duration). Therefore, more relevant studies will be needed to validate in the future.

## Conclusion

The current study suggests that T1DM patients with suboptimal glycemic control have a worse cognitive function, but only based on the scale of FSIQ. Moreover, a number of longitudinal studies are needed to further illuminate these present results. In this case, a comprehensive neuropsychological evaluation should be performed early in T1DM patients with poor glycemic control using standardized detection methods. This study highlights the importance of maintaining satisfactory glycemic control in patients with T1DM to improve their health status and quality of life. Future studies are needed to more precisely identify risk and protective factors for cognitive deficits in T1DM, and these results have the potential to influence patient treatment standards and guide treatment decisions.

### Supplementary Information

**Additional file 1.** PRISMA Checklist.

**Additional file 2.** Search strategy.

**Additional file 3:** **Table 1.** Risk of bias in the included studies. **Figure 1.** The prediction intervals for memory.

## Data Availability

Data were extracted from published sources.

## Abbreviations

CNKI: China National Knowledge Infrastructure
CI: Confidence interval
DM: Diabetes mellitus
FSIQ: Full-scale intellectual quotient
GRADE: Grading of Recommendations Assessment, Development and Evaluation
HbA1c: Glycated hemoglobin
IDF: International Diabetes Federation
JBI: Joanna Briggs Institute
MCI: Mild cognitive impairment
PROSPERO: International Prospective Register of Systematic Reviews
PI: Prediction interval
SMD: Standardized mean difference
STZ: Streptozotocin
T1DM: Type 1 diabetes mellitus
T2DM: Type 2 diabetes mellitus
VIQ: Verbal intellectual quotient
