# Supplementary material for: Effect of glycemic control on cognitive function in patients with type 1 diabetes mellitus: a systematic review and meta-analysis
Source: Syst Rev. 2024 Jan 2;13:10. doi: 10.1186/s13643-023-02433-9 (PMC10763190; doi:10.1186/s13643-023-02433-9)
Supplement: Supplementary file 2 — Additional file 2. Search strategy. [file 13643_2023_2433_MOESM2_ESM.docx]

Box1 PubMed searching strategy

**#1** "Diabetes Mellitus, Type 1"[Mesh]

**#2** ((((((((((((((((((((((((((Diabetes Mellitus, Insulin-Dependent[Title/Abstract]) OR (Diabetes Mellitus, Insulin Dependent[Title/Abstract])) OR (Insulin-Dependent Diabetes Mellitus[Title/Abstract])) OR (Diabetes Mellitus, Juvenile-Onset[Title/Abstract])) OR (Diabetes Mellitus, Juvenile Onset[Title/Abstract])) OR (Juvenile-Onset Diabetes Mellitus[Title/Abstract])) OR (IDDM[Title/Abstract])) OR (Juvenile-Onset Diabetes[Title/Abstract])) OR (Diabetes, Juvenile-Onset[Title/Abstract])) OR (Juvenile Onset Diabetes[Title/Abstract])) OR (Diabetes Mellitus, Sudden-Onset[Title/Abstract])) OR (Diabetes Mellitus, Sudden Onset[Title/Abstract])) OR (Sudden-Onset Diabetes Mellitus[Title/Abstract])) OR (Type 1 Diabetes Mellitus[Title/Abstract])) OR (Diabetes Mellitus, Insulin-Dependent, 1[Title/Abstract])) OR (Insulin-Dependent Diabetes Mellitus 1[Title/Abstract])) OR (Insulin Dependent Diabetes Mellitus 1[Title/Abstract])) OR (Type 1 Diabetes[Title/Abstract])) OR (Diabetes, Type 1[Title/Abstract])) OR (Diabetes Mellitus, Type 1[Title/Abstract])) OR (Diabetes, Autoimmune[Title/Abstract])) OR (Autoimmune Diabetes[Title/Abstract])) OR (Diabetes Mellitus, Brittle[Title/Abstract])) OR (Brittle Diabetes Mellitus[Title/Abstract])) OR (Diabetes Mellitus, Ketosis-Prone[Title/Abstract])) OR (Diabetes Mellitus, Ketosis Prone[Title/Abstract])) OR (Ketosis-Prone Diabetes Mellitus[Title/Abstract])

**#3** #1 OR #2

**#4** "Glycemic Control"[Mesh]

**#5** ((((Glycemic Control[Title/Abstract]) OR (Control, Glycemic[Title/Abstract])) OR (Blood Glucose Control[Title/Abstract])) OR (Control, Blood Glucose[Title/Abstract])) OR (Glucose Control, Blood[Title/Abstract])

**#6** #4 OR #5

**#7** ("Cognition"[Mesh]) OR "Cognition Disorders"[Mesh]

**#8** (((((((Cognition*[Title/Abstract]) OR (Cognition Disorders[Title/Abstract])) OR (Functions, Cognitive[Title/Abstract])) OR (Function, Cognitive[Title/Abstract])) OR (Cognitive Function*[Title/Abstract])) OR (Disorder, Cognition[Title/Abstract])) OR (Disorders, Cognition[Title/Abstract])) OR (Overinclusion[Title/Abstract])

**#9** #7 OR #8

**#10** #3 AND #6 AND #9

Box 2 Web of Science searching strategy

**#1** TS= (Diabetes Mellitus, Insulin-Dependent OR Diabetes Mellitus, Insulin Dependent OR Insulin-Dependent Diabetes Mellitus OR Diabetes Mellitus, Juvenile-Onset OR Diabetes Mellitus, Juvenile Onset OR Juvenile-Onset Diabetes Mellitus OR IDDM OR Juvenile-Onset Diabetes OR Diabetes, Juvenile-Onset OR Juvenile Onset Diabetes OR Diabetes Mellitus, Sudden-Onset OR Diabetes Mellitus, Sudden Onset OR Sudden-Onset Diabetes Mellitus OR Type 1 Diabetes Mellitus OR Diabetes Mellitus, Insulin-Dependent, 1 OR Insulin-Dependent Diabetes Mellitus 1 OR Insulin Dependent Diabetes Mellitus 1 OR Type 1 Diabetes OR Diabetes, Type 1 OR Diabetes Mellitus, Type 1 OR Diabetes, Autoimmune OR Autoimmune Diabetes OR Diabetes Mellitus, Brittle OR Brittle Diabetes Mellitus OR Diabetes Mellitus, Ketosis-Prone OR Diabetes Mellitus, Ketosis Prone OR Ketosis-Prone Diabetes Mellitus)

**#2** TS= (Control, Glycemic OR Blood Glucose Control OR Control, Blood Glucose OR Glucose Control, Blood OR Glycemic Control)

**#3** TS= (Cognition* OR Function*, Cognitive OR Cognitive Function* OR Disorder*, Cognition OR Overinclusion OR Cognition Disorders)

**#4** #1 AND #2 AND #3

Box 3 Cochrane Library searching strategy strategy

**#1** MeSH descriptor: [Diabetes Mellitus, Type 1] explode all trees

**#2** (Diabetes Mellitus, Insulin-Dependent OR Diabetes Mellitus, Insulin Dependent OR Insulin-Dependent Diabetes Mellitus OR Diabetes Mellitus, Juvenile-Onset OR Diabetes Mellitus, Juvenile Onset OR Juvenile-Onset Diabetes Mellitus OR IDDM OR Juvenile-Onset Diabetes OR Diabetes, Juvenile-Onset OR Juvenile Onset Diabetes OR Diabetes Mellitus, Sudden-Onset OR Diabetes Mellitus, Sudden Onset OR Sudden-Onset Diabetes Mellitus OR Type 1 Diabetes Mellitus OR Diabetes Mellitus, Insulin-Dependent, 1 OR Insulin-Dependent Diabetes Mellitus 1 OR Insulin Dependent Diabetes Mellitus 1 OR Type 1 Diabetes OR Diabetes, Type 1 OR Diabetes Mellitus, Type 1 OR Diabetes, Autoimmune OR Autoimmune Diabetes OR Diabetes Mellitus, Brittle OR Brittle Diabetes Mellitus OR Diabetes Mellitus, Ketosis-Prone OR Diabetes Mellitus, Ketosis Prone OR Ketosis-Prone Diabetes Mellitus):ti,ab,kw

**#3** #1 OR #2

**#4** MeSH descriptor: [Glycemic Control] explode all trees

**#5** (Control, Glycemic OR Blood Glucose Control OR Control, Blood Glucose OR Glucose Control, Blood OR Glycemic Control):ti,ab,kw

**#6** #4 OR #5

**#7** MeSH descriptor: [Cognition] explode all trees

**#8** MeSH descriptor: [Cognition Disorders] explode all trees

**#9**(Cognition* OR Function*, Cognitive OR Cognitive Function* OR Disorder*, Cognition OR Overinclusion OR Cognition Disorders):ti,ab,kw

**#10** #7 OR #8 OR #9

**#11** #3 AND #6 AND #10

Box 4 Embase searching strategy strategy strategy

**#1** 'insulin dependent diabetes mellitus'/exp

**#2** 'diabetes mellitus, insulin-dependent':ab,ti OR 'diabetes mellitus, insulin dependent':ab,ti OR 'insulin-dependent diabetes mellitus':ab,ti OR 'diabetes mellitus, juvenile-onset':ab,ti OR 'diabetes mellitus, juvenile onset':ab,ti OR 'juvenile-onset diabetes mellitus':ab,ti OR iddm:ab,ti OR 'juvenile-onset diabetes':ab,ti OR 'diabetes, juvenile-onset':ab,ti OR 'juvenile onset diabetes':ab,ti OR 'diabetes mellitus, sudden-onset':ab,ti OR 'diabetes mellitus, sudden onset':ab,ti OR 'sudden-onset diabetes mellitus':ab,ti OR 'type 1 diabetes mellitus':ab,ti OR 'diabetes mellitus, insulin-dependent, 1':ab,ti OR 'insulin-dependent diabetes mellitus 1':ab,ti OR 'insulin dependent diabetes mellitus 1':ab,ti OR 'type 1 diabetes':ab,ti OR 'diabetes, type 1':ab,ti OR 'diabetes mellitus, type 1':ab,ti OR 'diabetes, autoimmune':ab,ti OR 'autoimmune diabetes':ab,ti OR 'diabetes mellitus, brittle':ab,ti OR 'brittle diabetes mellitus':ab,ti OR 'diabetes mellitus, ketosis-prone':ab,ti OR 'diabetes mellitus, ketosis prone':ab,ti OR 'ketosis-prone diabetes mellitus':ab,ti

**#3** #1 OR #2

**#4** 'glycemic control'/exp

**#5** 'control, glycemic':ab,ti OR 'blood glucose control':ab,ti OR 'control, blood glucose':ab,ti OR 'glucose control, blood':ab,ti OR 'glycemic control':ab,ti

**#6** #4 OR #5

**#7** 'cognition'/exp

**#8** 'cognitive defect'/exp

**#9** cognition*:ab,ti OR 'function*, cognitive':ab,ti OR 'cognitive function*':ab,ti OR 'disorder*, cognition':ab,ti OR overinclusion:ab,ti OR 'cognitive defect':ab,ti

**#10** #7 OR #8 OR #10

**#11** #3 AND #6 AND #10
