# Supplementary material for: Effect of glycemic control on cognitive function in patients with type 1 diabetes mellitus: a systematic review and meta-analysis
Source: Syst Rev. 2024 Jan 2;13:10. doi: 10.1186/s13643-023-02433-9 (PMC10763190; doi:10.1186/s13643-023-02433-9)
Supplement: Supplementary file 3 — Additional file 3: Table 1. Risk of bias in the included studies. Figure 1. The prediction intervals for memory. [file 13643_2023_2433_MOESM3_ESM.docx]

**Table 1** Risk of bias in the included studies.

| **Study** | **Item 1** | **Item 2** | **Item 3** | **Item 4** | **Item 5** | **Item 6** | **Item 7** | **Item 8** |
| --- | --- | --- | --- | --- | --- | --- | --- | --- |
| Ohmann 2009 | low risk | low risk | low risk | low risk | low risk | low risk | unclear | low risk |
| Zihl 2010 | low risk | high risk | low risk | low risk | high risk | high risk | unclear | high risk |
| Kaufmann 2012 | low risk | low risk | low risk | low risk | low risk | low risk | unclear | low risk |
| Abo-el-Asrar 2016 | low risk | low risk | low risk | low risk | high risk | high risk | unclear | low risk |
| HE 2018 | low risk | low risk | low risk | low risk | low risk | high risk | unclear | low risk |
| STANISŁAWSKA-KUBIAK 2018 | low risk | low risk | low risk | low risk | high risk | high risk | unclear | high risk |

Item 1, Were the criteria for inclusion in the sample clearly defined; Item 2, Were the study subjects and the setting described in detail; Item 3, Was the exposure measured in a valid and reliable way; Item 4, Were objective, standard criteria used for measurement of the condition; Item 5, Were confounding factors identified; Item 6, Were strategies to deal with confounding factors stated; Item 7, Were the outcomes measured in a valid and reliable way; Item 8, Was appropriate statistical analysis used.


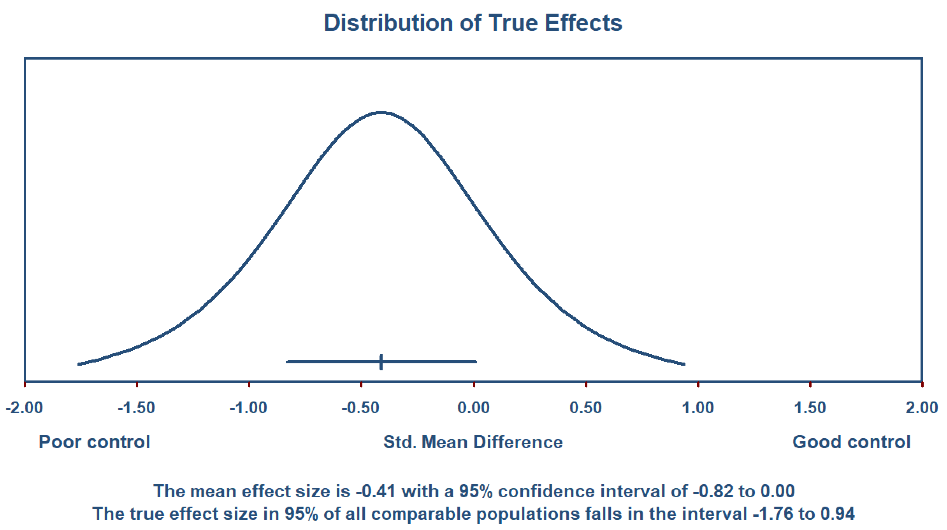


**Fig. 1** The prediction intervals for memory.
